# Remarks on Twenty-Five Cases of Splenotomy

**Published:** 1879-12

**Authors:** 


### SELECTIONS.

---

### REMARKS ON TWENTY-FIVE CASES OF SPLENOTOMY.

[In connection with Dr. J. F. Miner's recent operation, for removal of an enlarged spleen, an article by Prof. Czerney, translated by Prof. A. Barkaw, M. D., will undoubtedly be interesting to many of the readers of the JOURNAL; we therefore copy it from the *Pacific Medical and Surgical Journal.*—ED.]

PROF. CZERNEY, who succeeded the late Simon in the chair of surgery, at the University of Heidelberg, and may be considered as one of the foremost of young German surgeons, has recently published a monograph on laparo-splenotomy, which contains the history of two cases operated on by himself, and some interesting remarks regarding the operation itself. Of twenty-five cases on record, six recovered (Zaccarelli, Forrerius, Pean (2), Martin, Czerney). Speaking of the condition of the patients, whose state of health was shown for a long period after the successfully performed operations, Czerney remarks: "The cases again prove the well-known fact that man is able to live without a spleen, without his functions undergoing an essential disturbance. The changes in the constitution of the blood are of such trifling nature, and soon pass away so completely, that they may be considered simply as caused by the operative proceeding. The passing swelling of the limph-glands does not seem either to be a constant sequel of excision of the spleen, nor is there a constant anomaly to be found as regards the digestion of the patients. Neither bread and butter, nor potatoes, agree with my patient; whilst to the second patient of Pean, meat is distasteful. His first patient, as well as Martin's case, had normal digestion. If, then, the spleen possesses the great significance in the pancreatic digestion, as Schiff supposes, it is replaced through supplemental organs, in a way similar to that in which the excluded stomachic digestion is supplanted in the living dog. A striking feature is the greatly excited nervous condition of these patients. As to the benefit conferred by the operation, there seems to be no doubt that in these four cases the trouble and dangers caused by the movable or enlarged and painful spleen have been lastingly removed.

"The diagnosis of splenic tumor may offer considerable difficulties as regards the indication; the migration of spleen (Undermilz), according to present experiences, undoubtedly, demands removal of that organ as soon as the symptoms grow to be very troublesome, and not to be removed by bandages. So far as leukemic tumors of the spleen are concerned, we will now show, after thirteen sad experiences, that we should be very cautious. The latter stages when a hemorrhagic diathesis, a highly cachetic condition, and an affection of the organs by the same disease, can be ascertained, are in the future to be excluded from an operation. The details of the operation can alone be decided by the results of the still rarely occurring cases of extirpation; the principles adopted in other abdominal operations, especially in ovariotomy, must be observed in laparo-splenotomy. I regard it as of the utmost importance to strictly enforce antiseptic rules: first, by using carbolic acid constantly but sufficiently, according to Lister's principles, to have an increased temperature of the rooms used for the operation, and to observe the general measures common in abdominal operation. Although surgeons of great experience, as Kuchler, Spencer Wells, and Ph. Bryant, make the abdominal section toward the side, yet I am in favor of Pean's cut in the median line. One has in that way the free field for action, as regards the possible presence of adhesions between the tumor and the concavity of the diaphragm. In such a case I would rather not give up the operation, but should consider it surgically justifiable to do as Tussenbauer did in his case of resection of the intestines, to make another incision through the abdominal walls, which, starting from the umbilicus at right angles with the median incision, should be carried into the left lumbar region.

"As to the second part of the operation, the drawing forth of the spleen from the abdominal cavity, Martin's case proves that one may meet with considerable difficulty with a small migrating spleen. Slight adhesions can easily be separated by the hand. The separation of firmer adhesions may easily lead to laceration of the splenic tissue. I have, therefore, in my second case, first ligated the vessels in situ, and only after that detached the adhesions. Notwithstanding that the hemorrhagic diathesis being present, death ensued from loss of blood from the torn blood-vessel. If, therefore, the abdominal cavity has been opened at all in a case where there is hemorrhagic diathesis, and firm adhesions of the spleen are found, one will act wisely, in following Nedopil's advice, to close the abdominal cavity again. It is best not to open it at all where there is such a condition present.

If the spleen has been delivered from the abdominal cavity, one must take care not to pull the pedicle too forcibly, as the thin-coated veins may easily be torn. For this reason I do not think a torsion of the pedicle (which caused the tearing of strong vein in Spencer Wells' first case), or the application of an ecraseur, advisable. Pean's second fortunate case, however, proves that, with great care, even an ecraseur may be used successfully. Nevertheless, it is my opinion that the best treatment of the pedicle consists in the ligature of the gastro-splenic ligament in mass, using well-disinfected silk, and sinking the shortly cut ligatures.

" Martin and myself were successful with this method. Pean also sinks, in his first case, the pedicle, which he had tied with four ligatures of silver wire. Martin correctly remarks that the extraperitoneal treatment of the pedicle is objectionable, because the traction of the stomach and large blood-vessels of the pedicle may lead to disagreeable consequences. If the silk which is to be used for ligature is previously coiled for half an hour in carbolated water, then cleansed in carbolated oil, it heals in the abdominal cavity without reaction. I have some reason to be pleased with this way of disposing of the pedicle in my ovariotomies. It depends upon the width of the pedicle in how many portions the filus vessels should be ligated. If, previous to detaching the spleen, all of the blood-vessels of the pedicle cannot be ligated, one must also apply peripheric ligatures, in order to prevent loss of blood from the lienal veins. If the pedicle be very short, I should not hesitate, as Billroth has proposed, to get the tail of the pancreas into a ligature. Martin recommends in such a case, rather to leave behind a (button) part of the lienal tissue, in order to prevent the sliding off of the ligature. The treatment of the other adhesions, the management of the peritoneum, the abdominal suture, and after treatment, differ in no way from the same process in ovariotomy.

